# Individual Analyses of Lévy Walk in Semi-Free Ranging Tonkean Macaques (*Macaca tonkeana)*

**DOI:** 10.1371/journal.pone.0026788

**Published:** 2011-10-26

**Authors:** Cédric Sueur, Léa Briard, Odile Petit

**Affiliations:** 1 Unit of Social Ecology, Free University of Brussels, Brussels, Belgium; 2 Centre National de la Recherche Scientifique, Département Ecologie, Physiologie et Ethologie, Strasbourg, France; 3 Université de Strasbourg, Institut Pluridisciplinaire Hubert Curien, Strasbourg, France; Texas A&M University, United States of America

## Abstract

Animals adapt their movement patterns to their environment in order to maximize their efficiency when searching for food. The Lévy walk and the Brownian walk are two types of random movement found in different species. Studies have shown that these random movements can switch from a Brownian to a Lévy walk according to the size distribution of food patches. However no study to date has analysed how characteristics such as sex, age, dominance or body mass affect the movement patterns of an individual. In this study we used the maximum likelihood method to examine the nature of the distribution of step lengths and waiting times and assessed how these distributions are influenced by the age and the sex of group members in a semi free-ranging group of ten Tonkean macaques. Individuals highly differed in their activity budget and in their movement patterns. We found an effect of age and sex of individuals on the power distribution of their step lengths and of their waiting times. The males and old individuals displayed a higher proportion of longer trajectories than females and young ones. As regards waiting times, females and old individuals displayed higher rates of long stationary periods than males and young individuals. These movement patterns resembling random walks can probably be explained by the animals moving from one location to other known locations. The power distribution of step lengths might be due to a power distribution of food patches in the enclosure while the power distribution of waiting times might be due to the power distribution of the patch sizes.

## Introduction

All animals have to adapt their activity and movements patterns to their environment in order to maximize their nutrient intakes and therefore their fitness [1]–[4]. How the heterogeneity of resource distribution and how it affects individual foraging decisions is of great interest in ecology [5], [6] but also in decision sciences [2], [7] and physics [8], [9]. Indeed, several species seem to behave like particles suspended in a fluid: they move in their environment according to Brownian motion [6], [10]–[12]. Authors have suggested that the use of these random walks should increase the probability of the subject to find food or a reproductive partner [10]–[15].

Lévy walks are a special class of random walks in which movement displacements (step lengths) and stationary periods (waiting times) are not constant but follow a probability distribution with a power-law tail [6], [16]. The opposite is seen in the Brownian walk, where the probability of showing a certain step length is constant, and its distribution follows an exponential law [11], [13], [17]. The topic continues to be discussed and questioned [5], [10], [17]–[19], but the main hypothesis is that the Lévy walk is used by animals as an optimal strategy when they are seeking resources without information about the heterogeneous environment with low density food patches in which they live. Previous studies have reported Lévy walks in social amoebas [20], zooplankton [10], jackals [16], albatrosses [6] and primates [19], [21]. However, few studies have assessed whether individual characteristics, such as body mass, age or sex could influence movement patterns. One study alone showed that Lévy walks differed between male and female spider monkeys (*Ateles geoffroyi*) [21]. Authors suggested that this difference was due to a different space-use strategy, with males ranging over wider areas than females in order to control the boundaries of their home range. Studying on an individual level rather than a population level is an interesting possibility to further develop our understanding of the emergence of random walks in animals. Here, we assess whether individuals from a semi free-ranging group of Tonkean macaques (*Macaca tonkeana*) show different movement patterns and whether these differences depend on socio-demographic variables.

Primates are known to use high-level cognitive processes in their foraging and movement decisions [7]. It would therefore be logical to consider that primates would not walk “randomly” (i.e. in an unpredictable way) in their environment. However, their physiological differences might affect how they will move, the distance traveled and the time they spend foraging, and therefore influence the distribution of their step lengths or waiting times. Differences between individuals had already been shown in several species, namely in diet and activity budget [22]–[30]. Resting tends to increase with age whilst moving decreases [22], [29]. In many cases, adult females spend more time foraging than adult males, but the opposite case can also be seen. This result mainly depends on the sexual dimorphism existing between the two genders, with adult males foraging more than females when the dimorphism increases [22], [23], [31]. In the same way, and due to their small body mass, young individuals spend less time foraging than adult ones. Finally, young individuals socialize, and particularly play more than adult individuals [32]. All these results showed that socio-demographic characteristics have a significant impact on how individuals adapt their activity to their environment.

We studied activity budget and movement patterns of ten Tonkean macaques living in a park containing grass, trees and bushes. Despite these semi-free ranging conditions, animals spend more than a third of their time foraging and searching for food (pellets provided within the indoor housing, as well as natural fruits, leaves and buds in their captive environment; see [33]). They also adjust their behaviour according to variation in environmental constraints throughout the day and in the course of a year [33], [34]. We first studied whether animals displayed differences in their activity budget, as already shown in several other primate species. We then used the maximum likelihood method to analyse the distribution of step lengths and waiting times for each individual [10], [19], [35]. This is considered to be the most efficient method when testing for the presence of Lévy walk in animals. We expected animals to display differences according to their age and their sex. For the distribution of step lengths, given the fact that young individuals play more (short movements) but forage less (long movements) than adults, we suggested that the tail of the power function would be longer in adults than in young individuals, i.e. that adults would walk less but would cover longer distances than juveniles. Given the sexual dimorphism between male and female Tonkean macaques, we suggested that males would forage more than females, and that the tail of the power function would be also longer for males. As adults rest more but play less than young individuals, we expected a longer tail of waiting time distribution in adults than in young individuals. We did not expect to see any difference in waiting time distribution between different genders.

## Materials and Methods

### Ethics Statement

This study involved the observation of animals without animal handling or invasive experiments carried out on studied subjects. The body masses of individuals were scored during a health check, performed during the observation period. We declare that our study was approved by our institution and carried out in full accordance with the ethical guidelines set out by this institution (certificate number: 67–339, French Republic, Bas-Rhin County Hall, French veterinary services). Our experiments comply with European animal welfare legislation. Please see the section below (*Subjects and environment*) for amelioration of animal welfare. The work being carried out during this study is in accordance with the weatherall report and all efforts were made to ensure the welfare of the animals and minimize suffering.

### Subjects and environment

The study group was bred under semi free-ranging conditions at the Strasbourg University Centre of Primatology. At the time of the study (November 2005 to March 2006), the group consisted of 10 individuals: one adult male (over five years old), five adult females (over four years old) and four juveniles (aged one to three years). This group composition is comparable to that of wild groups [33], [36]–[38]. Wild Tonkean macaques are typically found in the primary and secondary rainforests of Sulawesi (Indonesia) and are mainly frugivorous [36], [38]. The Tonkean macaques in this study had complete access to 0.32 ha (maximal length = 80 m; maximal width = 60 m) of wooded parkland as well as indoor housing within the enclosure. The indoor housing (20 m^2^) is made of cement and tiling, and animals were able to climb on it. The enclosure area was made up of various slopes and uneven ground. The distribution of vegetation was also heterogeneous, with three layers (grass, trees and bushes) that were unevenly distributed throughout the enclosure. Within the park, animals moved cohesively during 50% of movements, in sub-groups in 20% of movements, and alone in 30% of movements [39]. They used the park in a heterogeneous way according to ecological conditions (topology and vegetation; [33]). Despite the *ad libitum* provision of commercial primate pellets and water within the indoor housing, animals were seen to spend 36.3% of their time foraging and searching for other food than the pellets provided (leaves, buds and fruits) outside the indoor enclosure (see [33] for details). Fresh fruit and vegetables were provided at the same location once a week, one hour after the end of the observation session. Thus, the behaviour of the animals was unlikely to be affected by this event. Animals were used to human presence in their enclosure.

### Scoring of variables

Observations occurred from November 18th, 2005 to March 23rd, 2006, for four hours per day, from 9:00 to 13:00 or from 13:00 to 17:00.

Every 10 minutes, an observer noted the position of each animal in the enclosure on a map (scale: 1/550; precision: one meter) and recorded each animal's activity, using the instantaneous sampling method [40]. The measurement of 77 landmarks enabled us to create a precise grid of the park with 1 m^2^ cells. The activities we were interested in (listed below) were defined according to the same criteria used in previous studies for Tonkean macaques [33], [36], [38]:

- **Moving**: locomotion including walking, running, climbing and jumping;
- **Foraging**: reaching for, picking, manipulating, masticating, or placing food in mouth, as well as manipulating the contents of a cheek pouch (if an individual masticated whilst walking, we considered it to be moving);
- **Resting**: Body stationary, usually sitting or lying down;
- **Social activities**: playing, grooming, sexual and aggressive behaviour.

We only retained scans where the position and the activity of all individuals could be noted. At the end of the study, we had obtained a total of 24 days of observation, 558 scans and 5580 recordings of individual positions within the enclosure.

We established the dominance relationships through the scoring of spontaneous agonistic interactions and drinking competition around a single source of orange juice. Individuals were then ranked in a matrix of agonistic interactions and the linearity of this ranking was checked using Matman software (h′ = 0.75, p = 0.0006; [41]).

### Data analysis

#### Parameters

We estimated the activity budget of each individual by determining the percentage of observations of each activity (number of scans spent in one activity divided by the total number of scans).

A step was defined as an interval in which either or both coordinates in two consecutive samples differed. The maps on which we scored spatial positions were used to directly calculate the length L of a step in meters for each individual. Waiting times were calculated for each individual using the number of samples in which an individual had not changed position. We did not consider the turning angle distributions in the current paper, as turning angles do not give any clear information relating to the hypotheses. Indeed, we were unable to predict how age or sex could influence turning angles of individuals.

#### Analyses

Individual characteristics are given in Table 1. As body mass and dominance rank are highly correlated to age of individuals (body mass: rs = 0.98, N = 10, p<0.00001; dominance rank: rs = −0.98, N = 10, p<0.00001), we performed correlation analyses with activity budget and movement patterns using age. Dominance rank is also correlated to body mass (rs = 1.00, N = 10, p<0.00001). We found no difference according to age between males and females (U = 7.00, N_males_ = 4, N_females_ = 6, p = 0.352, m_males_ = 3[2]; [9], m_females_ = 7 [5]; [10]).

**Table 1:** Individual characteristics of Tonkean macaques.

ID	Age	Body mass (kg)	Dominance rank	Sex
Ga	11	14.8	1	M
Je	12	12.7	2	F
La	10	9.6	3	F
Ne	8	9.4	4	F
Ol	7	9	5	F
Pa	6	8.2	6	F
Sh	4	7	7	M
Ta	2	6.5	8	M
Ul	2	6.1	9	M
Uj	2	4.5	10	F

Firstly, we used Spearman rank correlation tests to correlate age with the absolute frequency of observations per activity per individual. We then tested the effect on activity using a Mann-Whitney test.

We checked for the predominance of either a Lévy walk (power distribution, y = a*x^μ^) or a Brownian walk (exponential distribution, y = a.e^x*λ^) in animals. This was achieved via the maximum likelihood method [10], [35]. The maximum likelihood method (MLE) involves calculating the exponent of the distribution (i.e. power and exponential in the case of the current study) in order to calculate the distribution log likelihood. Log likelihoods for the exponential distribution and the power distribution can then be compared for step lengths and waiting times using the Akaike Information Criterion (AIC). One AIC is calculated for each hypothesis (Brownian or Lévy) and we retained the hypothesis with the lowest AIC.

For the step length distribution, we first calculated the maximum likelihood estimate of the power law exponent as follows:
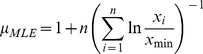
(1)Where *n* is the total number of step lengths, *x_min_* is the minimum step length included in the analysis (in the current study, the minimum measured step length equals one meter). Considering the maximum likelihood estimate, we can now calculate the log likelihood for the power law as:
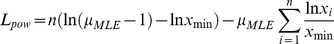
(2)The maximum estimate of the exponential law exponent was then calculated as:
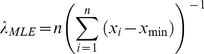
(3)And the log likelihood of the exponential distribution calculated as:
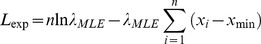
(4)Concerning the waiting times distribution, as the waiting time data are discrete, we changed the equation (5) with the following corrected formula [19]:
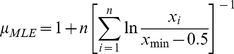
(5)We also changed the equation 3 to:
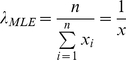
(6)In the current study, the minimum waiting time was 5 minutes. The log likelihoods for the waiting time distributions are calculated are calculated via equation 2 for the Lévy walk (power law) and via equation 4 for the Brownian walk (exponential law).

Using these measures, we then calculated the AIC of the two distributions (power and exponential) for both step length and waiting time:
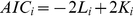
(7)where *K_i_* is the number of free parameters in the model *i* (in the current study, the exponent is the only parameter for both the power and the exponential distribution). We then calculated an Akaike weight *ω_i_* for each model *i* (Brownian and Lévy):
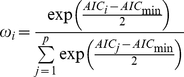
(8)This Akaike weight is dichotomised as scores of 0 for the unsuccessful model (highest AIC) and 1 for the successful model (lowest AIC) when comparing two models. In the current study, we checked which distribution *i* scored 1 in order to characterize the walk of animals as *i* (Brownian or Lévy).

A Kolmogorov-Smirnov test was used to test the uniformity of distributions for maximum step lengths and maximum waiting time (*x_max_*). We then correlated the estimates for exponents and log likelihood to age using Spearman rank correlation tests. Mann-Whitney tests were used to assess whether the estimates and log likelihoods differed between males and females.

The significance level was set at 0.05. We used the exact significance method for small sampling size. All tests were two-tailed. We carried out the analyses using SPSS 10.0 (SPSS Inc., Chicago, IL, USA). Values are presented as median and inter-quartiles.

## Results

### Activity budget

**Figure 1:**
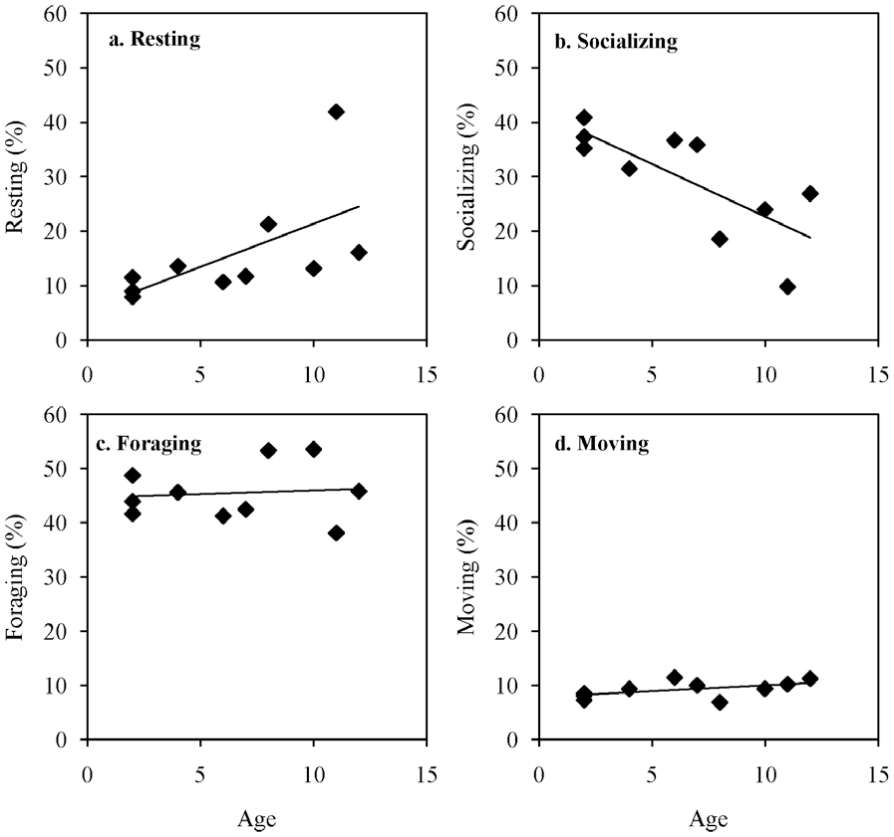
Percentage of observations for each activity (a. resting, b. socializing, c. foraging and d. moving) according to the ages of individuals.

Analyses showed that age is correlated with resting (rs = 0.80, N = 10, p = 0.005, fig. 1a) and socializing (rs = −0.78, N = 10, p = 0.008, fig. 1b) activities but not with foraging (rs = 0.12, N = 10, p = 0.748, fig. 1c) and moving activities (rs = 0.52, N = 10, p = 0.121, fig. 1c). The younger the individuals were, the less they were observed to rest and the more they were observed to socialize. More specifically, young individuals played together. Whatever the activity, no difference was observed between males and females (Table 2).

**Table 2:** Statistical and descriptive values of differences of activities between males and females.

		Activity			
		Resting	Socializing	Foraging	Moving
Statistics	Mann-Whitney U	11.00	9.00	5.00	9.50
	P-value	0.914	0.610	0.171	0.610
Males(N = 4)	Median	60	165	205	43
	Lower inter-quartile	46	73	187	36
	Upper inter-quartile	167	192	217	48
Females(N = 6)	Median	59	149	227	46
	Lower inter-quartile	48	108	202	37
	Upper inter-quartile	83	173	256	54

The descriptive values are the number of observations in each activity.

### Distribution of step lengths

**Figure 2:**
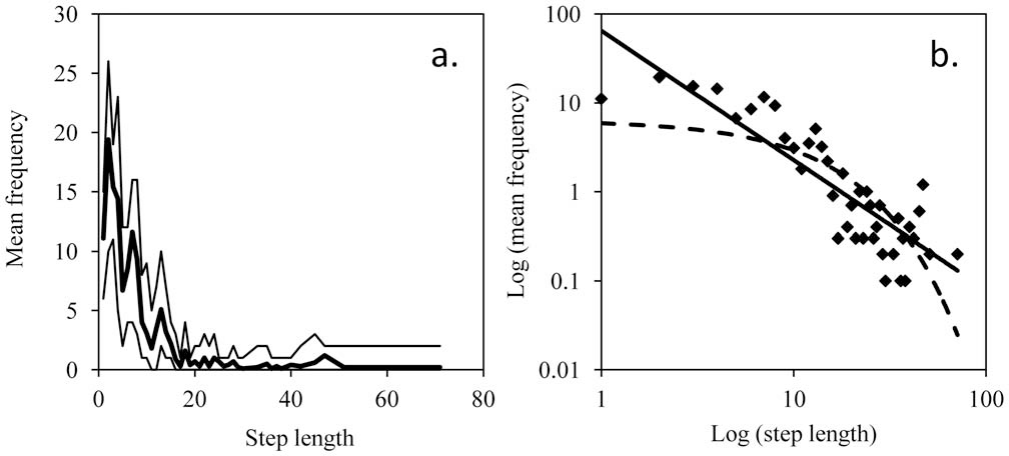
Distribution (mean frequency) of step lengths (meters). (a.) Normal distribution. The bold line is the mean frequency among all group members. The upper thin line is the maximum observed in the group. The lower thin line is the minimum observed in the group. (b.) Log-log distribution. The continuous line is the best-fitting power curve. The dotted line is the best-fitting exponential curve.

Information concerning the maximum likelihood estimates of both exponential and power distributions for each individual are given in Table 3. The Akaike weight for the power distribution equalled 1 for each individual (AIC_pow_ is the lowest AIC) meaning that all individuals seemed to follow a Lévy walk rather than a Brownian one (see Fig. 2 for a graphical representation).

**Table 3:** Information concerning the maximum likelihood estimates for power and exponential distributions of step lengths.

	n	μ_mle_	L_pow_	λ_mle_	L_exp_	AIC_pow_	AIC_exp_	ω_pow_	ω_exp_
Néréis	92	1,63	−279,91	60858	−40256553,5	562	80513109	1	0
Shan	176	1,57	−587,90	221584	−278972090	1178	557944181	1	0
Gaetan	125	1,56	−421,54	120375	−115919663	845	231839327	1	0
Tao	143	1,63	−435,45	116259	−94516899,1	873	189033800	1	0
Ulysse	164	1,62	−509,10	163836	−163670195	1020	327340392	1	0
Jeanne	96	1,74	−255,27	46656	−22673783,9	513	45347569,9	1	0
Patsy	139	1,56	−470,69	162352	−189625468	943	379250939	1	0
Olga	137	1,64	−410,36	111792	−91220679,5	823	182441361	1	0
Ujung	144	1,66	−419,88	116064	−93545904,7	842	187091811	1	0
Lady	98	1,63	−297,93	63798	−41531413,8	598	83062829,6	1	0

We then performed analyses on the power estimates for the step length distributions.

Age is correlated to the log likelihood of the power law *L_pow_* (rs = 0.67, N = 10, p = 0.034) but not to the exponent *μ_MLE_* (rs = 0.18, N = 10, p = 0.623). The dominant male (with the highest body mass) displays the longest tailed distribution of step lengths. Indeed, his maximum step length was 71 meters (non-uniform distribution with other group members: Z = 1.98, p = 0.001) whilst maximum step lengths in other group members ranged from 42 to 51 meters (m_maximum_ = 47[46.5;48], uniform distribution: Z = 1.00, p = 0.270). The log likelihood of the power law *L_pow_* differed between males and females (U = 2.00, N_males_ = 4, N_females_ = 6, p = 0.033, m_males_ = −472 [−528; −425], m_females_ = −354[−432; −273]) whereas the exponent *μ_MLE_* did not (U = 4.00, N_males_ = 4, N_females_ = 6, p = 0.088, m_males_ = 1.59 [1.56;1.63], m_females_ = 1.64[1.61;1.68]) even if a tendency is visible.

### Waiting time distributions

**Figure 3:**
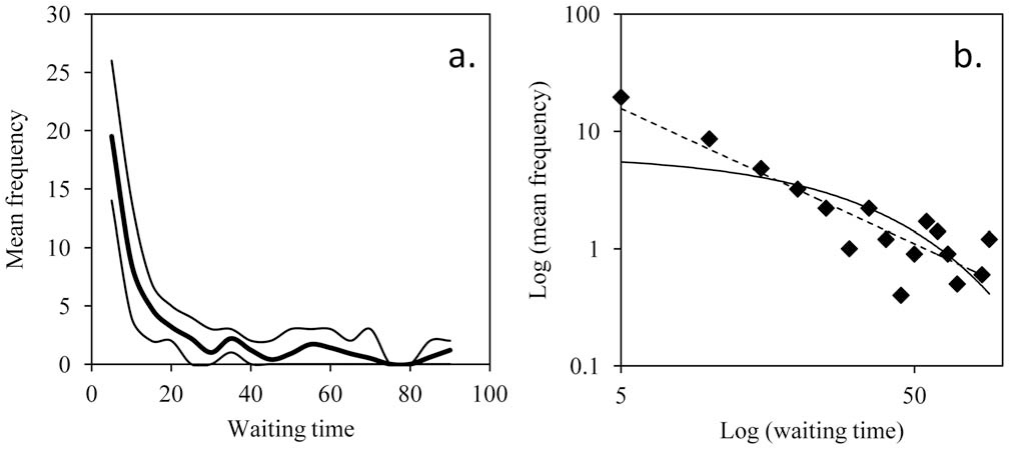
Distribution (mean frequency) of waiting times (minutes). (a.) Normal distribution. The bold line is the mean frequency among all group members. The upper thin line is the maximum observed in the group. The lower thin line is the minimum observed in the group. (b.) Log-log distribution. The continuous line is the best-fitting power curve. The dotted line is the best-fitting exponential curve.

Information concerning the maximum likelihood estimates of both exponential and power distributions for each individual are given in Table 4. The Akaike weight for the power distribution equalled 1 for each individual (AIC_pow_ is the lowest AIC) meaning that all individuals seemed to follow a Lévy walk more than a Brownian one (see Fig. 3 for a graphical representation).

**Table 4:** Information concerning the maximum likelihood estimates for the power and exponential distributions of waiting times.

	n	umle	lpow	λmle	lexp	AICpow	AICexp	wpow	wexp
Néréis	35	1,58	−114,32	0,03	−156,16	231	314	1	0
Shan	57	2,28	−87,46	0,07	−201,57	177	405	1	0
Gaetan	57	2,06	−107,10	0,06	−218,53	216	439	1	0
Tao	51	2,11	−91,23	0,05	−198,54	184	399	1	0
Ulysse	39	1,95	−81,75	0,05	−158,11	165	318	1	0
Jeanne	52	1,85	−121,45	0,04	−213,19	245	428	1	0
Patsy	47	1,93	−100,99	0,05	−188,00	204	378	1	0
Olga	50	1,96	−104,42	0,05	−197,29	211	397	1	0
Ujung	46	1,94	−97,87	0,05	−184,55	198	371	1	0
Lady	54	1,84	−127,74	0,05	−218,56	257	439	1	0

We then performed analyses on the power estimates for the waiting time distributions.

Age is correlated to the log likelihood of the power law *L_pow_* (rs = −0.88, N = 10, p = 0.001) but not to the exponent *μ_MLE_* (rs = −0.44, N = 10, p = 0.208). The exponent *μ_MLE_* of the power law differed between males and females (U = 1.00, N_males_ = 4, N_females_ = 6, p = 0.019, m_males_ = −89 [−103; −83], m_females_ = −109[−123; −100]) whereas the log likelihood *L_pow_* did not, even if a tendency is visible (U = 3.00, N_males_ = 4, N_females_ = 6, p = 0.055_males_ = 2 [1.98;2.23], m_females_ = 1.89[1.77;1.94]). Maximum waiting times ranged from 60 to 90 and the median is 90 [81.25;90]. The distribution of maximum waiting time is not uniform between individuals (Z = 2.00, p = 0.01).

## Discussion

This is the first study to examine the possible effects of socio-demographic variables on the individual distributions of step lengths and waiting times. The results are as we expected. Individuals differed in their activity budget and in their movement patterns. We found an effect of age and sex of individuals on the power distribution of their step lengths and of their waiting times.

The Lévy walk has been described as an optimal strategy for food research efficiency or reproduction in several species [11], [13]. This link between the Lévy walk and optimality can be applied when the forager lives in a heterogeneous environment with low-density food patches and has no information concerning the location of food supplies [6], [11]. For instance, marine predators switch between Lévy and Brownian movement as they move through different types of habitat, the Brownian walk being associated with the presence of abundant prey [11]. More questions arise about the significance of Lévy walks when this so-called “random” movement - implying that it is “unpredictable”- is found in species which possess high cognitive skills and a mental map of their environment, such as the primates in the current study [17], [19], [42]. In this context, the criteria justifying a Lévy walk are no longer applicable, even if some authors [21] have suggested that a Lévy walk may favour the ripening of fruit or leaves before the next visit to the patch in question. In animals that are informed about their environment and have a cognitive map of where they are living, the hypothesis of a random walk is no longer sustainable. Finding a walk which ressembles a Brownian or Lévy walk is therefore due to the distance distribution between the different spots (food patches, waterhole, resting sites) the animals visit. For instance, Ramos-Fernández, Boyer and colleagues explain that the Lévy walk found in spider monkeys was directly dependent on the environment in which animals lived. They built a model in which the monkeys follow mental maps and chose to move according to a maximum efficiency criterion in a spatially disordered environment containing trees with a heterogeneous size distribution. They showed that moving the tree size frequency distribution could result in different random walks such as the Lévy walk [18], [42], [43]. Schreier and Grove could not distinguish whether another species of primates, the hamadryas baboon (*Papio hamadryas*), displayed Brownian or Lévy walks. They also attributed their findings to the distribution of food patches in the baboon environment [19]. In the current study, we were able to identify whether Tonkean macaques displayed a Lévy walk. It is interesting to note that the subjects still displayed a Lévy walk in a semi-free ranging environment where food was given *ad libitum*. These movement patterns resembling random walks can probably be explained by the animals moving from one location to other known locations. The power distribution of step lengths might be due to a power distribution of food patches in the enclosure whilst the power distribution of waiting times might be due to the power distribution of the patch sizes. For instance, animals spend a higher quantity of time in their indoor housing where pellets and water are *ad libitum*.

Even if movement patterns depend directly on the environment, animals should still display differences in step length or waiting times distribution, since they do not have the same nutrient or social requirements [44]. Previous studies on different species had already found differences in activities according to the sex or age of individuals [22], [25], [29]–[31], [45]. In this study, we found that age has an influence on the time individuals will spend resting or socializing. Young individuals rested less but socialized more than older individuals. We predicted that individual differences also have a direct impact on the movement patterns of individuals. We consequently found an effect of age and sex on the distribution of step lengths and waiting times in the Tonkean macaques we studied. The males and old individuals displayed a higher proportion of longer trajectories than females and young ones. As regards waiting times, females and old individuals displayed higher rates of long stationary periods than males and young individuals. The difference between males and females has already been described in spider monkeys, and authors suggested that the specific activities of males such as controlling group home range boundaries might lead to this difference [21] because the sexual dimorphism in spider monkeys is not very high. In the current study, macaques displayed great differences in body mass according to their age and to their sex. In addition to their social difference (group members need to socialize when they are juveniles), this physiological difference (in terms of physiological needs per day) directly results in different patterns of movements. The maximum step length of the dominant male - the individual with the highest body mass - also seems to be different to that observed in its conspecifics. One can suggest that the dominant male shows the longest trajectories because it is the top-ranking individual and as such, probably expects the rest to follow him, whereas other individuals would negotiate about the direction to take. However, we showed that Tonkean macaques displayed an equally-shared consensus where everybody, irrelevant of social status, can initiate a movement and be followed by the entire group [34], [39].

The Lévy walk has been described as an optimal strategy for individuals searching for food, but has always been studied without taking the characteristics of each individual into account. This study is the first to thoroughly analyse the influence of socio-demographic variables on the movement patterns of individuals. In spite of the semi-free ranging conditions, we found clear differences between individuals and we have suggested different explanations for these results. The next step would be to investigate the influence of individual variables on movement patterns in other species - i.e. from solitary to social - in order to better understand how the Lévy walk emerges and to confirm its evolutionary significance.
